# Forced Ambiguity of the Leucine Codons for Multiple-Site-Specific Incorporation of a Noncanonical Amino Acid

**DOI:** 10.1371/journal.pone.0152826

**Published:** 2016-03-30

**Authors:** Inchan Kwon, Eun Sil Choi

**Affiliations:** 1 School of Materials Science and Engineering, Gwangju Institute of Science and Technology (GIST), Gwangju, Republic of Korea; 2 Department of Chemical Engineering, University of Virginia, Charlottesville, Virginia, United States of America; 3 Department of Biological Sciences, College of Natural Sciences, Chonnam National University, Gwangju, Republic of Korea; Berlin Institute of Technology, GERMANY

## Abstract

Multiple-site-specific incorporation of a noncanonical amino acid into a recombinant protein would be a very useful technique to generate multiple chemical handles for bioconjugation and multivalent binding sites for the enhanced interaction. Previously combination of a mutant yeast phenylalanyl-tRNA synthetase variant and the yeast phenylalanyl-tRNA containing the AAA anticodon was used to incorporate a noncanonical amino acid into multiple UUU phenylalanine (Phe) codons in a site-specific manner. However, due to the less selective codon recognition of the AAA anticodon, there was significant misincorporation of a noncanonical amino acid into unwanted UUC Phe codons. To enhance codon selectivity, we explored degenerate leucine (Leu) codons instead of Phe degenerate codons. Combined use of the mutant yeast phenylalanyl-tRNA containing the CAA anticodon and the yPheRS_naph variant allowed incorporation of a phenylalanine analog, 2-naphthylalanine, into murine dihydrofolate reductase in response to multiple UUG Leu codons, but not to other Leu codon sites. Despite the moderate UUG codon occupancy by 2-naphthylalaine, these results successfully demonstrated that the concept of forced ambiguity of the genetic code can be achieved for the Leu codons, available for multiple-site-specific incorporation.

## Introduction

Site-specific incorporation of a noncanonical amino acid into a protein has been widely used to provide unique physical, chemical, or biological properties to a protein [1–10]. In most cases, a noncanonical amino acid was introduced into a single site of a target protein. An amber codon was most commonly used as an incorporation site, though other stop codons and four-base codons were also used [3, 11–14]. In order to expand the utility of site-specific incorporation of a noncanonical amino acid, researchers attempted to achieve site-specific incorporation at multiple sites [15–17]. A pre-requisite to achieve noncanonical amino acid incorporation at multiple sites is to develop a new codon that can be reassigned to a noncanonical amino acid(s). Combinations of stop codons and four-base codons have been successfully used to encode two different noncanonical amino acids, resulting in a protein with two different noncanonical amino acids at two programmed sites [15, 18]. In this suppression strategy, incorporation of each noncanonical amino acid requires the expression of a corresponding heterologous orthogonal pair in host cells. Due to the limited number of orthogonal pairs available, it is challenging to incorporate noncanonical amino acids into more than two sites with the suppression strategy.

In order to circumvent this limitation, several groups achieved site-specific incorporation of a single noncanonical amino acid into multiple sites [19, 20]. We can easily imagine many situations where site-specific incorporation of a single amino acid into multiple sites is required. For instance, conjugation of multiple poly(ethylene glycol) molecules to reactive noncanonical amino acids of a therapeutic protein is expected to be more effective in enhancing the serum half-life than single poly(ethylene glycol) molecule conjugation. Recently, incorporation of p-azidophenylalanine into several sites was achieved using engineered *E*. *coli* cells of which all amber codons in genomic DNA were mutated into other stop codons and the release factor 1 gene was removed from genomic DNA to enhance amber suppression efficiency [19]. *In vivo* evolution of aaRS in the *E*. *coli* genome further enhanced the incorporation efficiency of Phe analogs into multiple sites of a target protein [21]. Alternatively, the degeneracy of the genetic code was explored for multiple-site-specific noncanonical amino acid incorporation [20]. In nature, several canonical amino acids, such as valine, leucine, isoleucine, methionine, phenylalanine, tyrosine, and tryptophan, are expected to be late addition to the genetic code [22]. Evolutionary analyses revealed that inclusion of methionine and tryptophan required the complete breaking of the codon wobble degeneracy [23]. Very recently it was reported that all tryptophan codons from *E*. *coli* genome are efficiently reassigned to a noncanonical amino acid, L-β-(thieno[3,2-b]pyrrolyl)alanine [24]. These are all very good indications that the exploitation of the degeneracy in the genetic code could represent a promising route for the expansion of amino acids available for protein biosynthesis [25]. By breaking the degeneracy of the Phe codons, a phenylalanine analog, 2-naphthylalanine (2Nal), was incorporated into multiple UUU phenylalanine (Phe) codons [20]. In order to reassign a UUU codon to 2Nal, the anticodon of yeast phenylalanyl-tRNA was mutated from CUA to AAA to generate ytRNA^Phe^_AAA_. A mutant yeast phenylalanyl-tRNA synthetase (ytRNA_T415G) was co-expressed with ytRNA^Phe^_AAA_ in *E*. *coli* cells to incorporate 2Nal into a target protein. Since this latter method uses a sense codon, the number of incorporation sites is not restricted [20]. Since UUU codons were not completely occupied by 2Nal, strictly speaking, the degeneracy of the Phe codons was not completely broken, but forced ambiguity of the Phe codons was achieved. Later, a yPheRS variant with a higher specificity toward 2Nal (yPheRS_naph) was selected from high-throughput screening of yPheRS libraries [26]. However, application of this technique has been limited, partly because 2Nal was misincorporated at the unwanted sites (UUC Phe codons) due to the less selective codon recognition of the AAA anticodon of ytRNA^Phe^_AAA_ [20]. Misincorporation of a noncanonical amino acid at unwanted sites might cause severe perturbation or loss of native properties of a target protein [27, 28].

Due to the poor discrimination of UUU codon from UUC codon by the AAA anticodon of ytRNA^Phe^_AAA_, we explored degenerate leucine (Leu) codons for noncanonical amino acid incorporation in this study. Several considerations recommend degenerate Leu codons. First, Leu is encoded as six codons: UUA, UUG, CUA, CUG, CUU, and CUC. Discrimination of UUG from CUN (N = A/U/G/C) codons should be highly efficient due to discrimination at the first position in the codon [29]. Second, our existing yeast orthogonal pair should be readily adapted to the incorporation of Phe analogs in response to UUG codons. In practical terms, generalization of the concept of forced ambiguity of the genetic code is limited by the availability of orthogonal pairs. Third, the modified CAA anticodon would more efficiently discriminate UUG from UUA. According to the wobble rules, C in the first position of the anticodon recognizes only G in the third position of the codon [29]. In this study, we first quantitatively evaluated the misincorporation level of 2Nal at unwanted sites (UUC codons) when the UUU Phe codon was reassigned to 2Nal. Then, we showed that an engineered ytRNA^Phe^_CAA_ containing a modified CAA anticodon could completely discriminate UUG from the remaining five Leu codons and achieve incorporation of 2Nal at multiple programmed sites in recombinant proteins.

## Results and Discussion

### Misincorporation of 2Nal at unwanted UUC Phe codons

**Fig 1:**
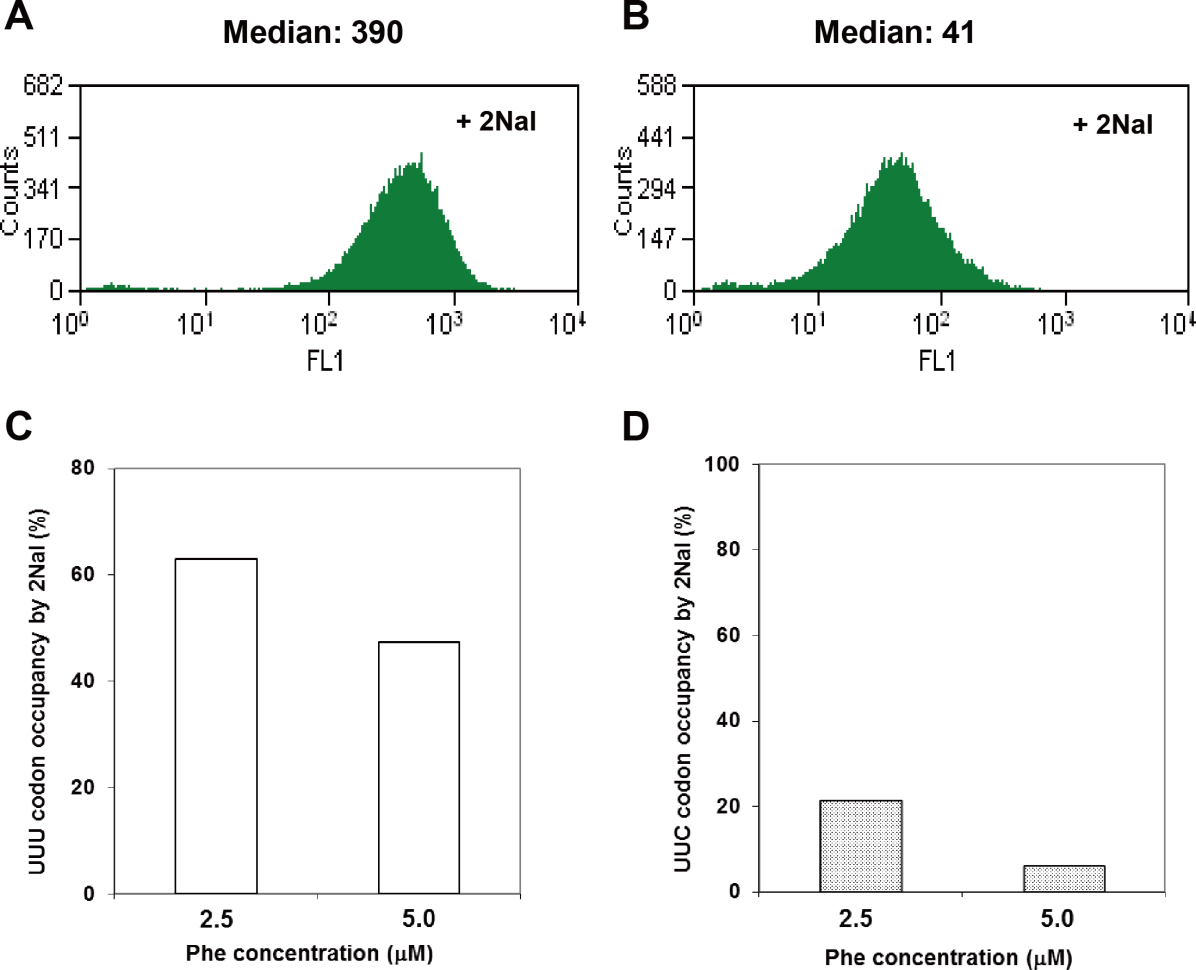
**Fluorescence intensities of cells expressing GFP6 variant (A and B).** GFP6 was expressed in DHF expression hosts outfitted with yPheRS_naph and ytRNA^Phe^_AAA_ in minimal medium supplemented with 18 amino acids, 5.0 μM Phe, 50 μM Trp, and no 2Nal (A); 3 mM 2Nal (B). UUC and UUU codon occupancy by Phe and 2Nal (C and D). Both GFP6 (2UUC) and GFP6 (2UUU) were expressed in DHF expression hosts outfitted with yPheRS_naph and ytRNA^Phe^_AAA_ in minimal medium supplemented with 18 amino acids (25 μg/mL), 50 μM Trp, 3 mM 2Nal, and either 2.5 μM or 5.0 μM Phe. The UUC (C) and UUU (D) codon occupancy by Phe and 2Nal were determined by N-terminal sequencing.

In order to evaluate the misincorporation level of 2Nal at UUC codons, we used a green fluorescent protein variant with Phe codons encoded by only the UUC codon (GFP6) by mutating all UUU Phe codons to UUC codons [26]. It was previously reported that 2Nal incorporation into multiple sites of GFP led to a significant loss of fluorescence due to the structural perturbation of GFP [26]. Since GFP6 does not have any 2Nal incorporation sites (UUU codon), little or no change in fluorescence intensity was expected, assuming 2Nal is not incorporated into UUC codons. However, in the presence of 3 mM 2Nal and 5 μM Phe, the mean fluorescence intensity of cells expressing GFP6 as well as ytRNA^Phe^_AAA_/yPheRS_naph decreased almost 10-fold compared to that of cells in the absence of 2Nal (Fig 1A and 1B). In the presence of 3 mM 2Nal and either 2.5 or 5.0 μM Phe, the occupancy of UUU and UUC codons by 2Nal was evaluated using liquid chromatography-tandem mass spectrometric (LC-MS/MS) analysis of tryptic digests of a mDHFR variant expressed in *E*. *coli* cells co-expressing ytRNA^Phe^_AAA_/yPheRS_naph (Fig 1C and 1D). At 5 μM Phe and 3 mM 2Nal, about 50% of the UUU codon was occupied by 2Nal, but 6% of the UUC codon was also occupied by 2Nal. Therefore, the reduction in cellular fluorescence of GFP6 expressed in the presence of 3 mM 2Nal (Fig 1B) could be attributed to misincorporation of 2Nal at multiple UUC codon sites.

**Fig 2:**
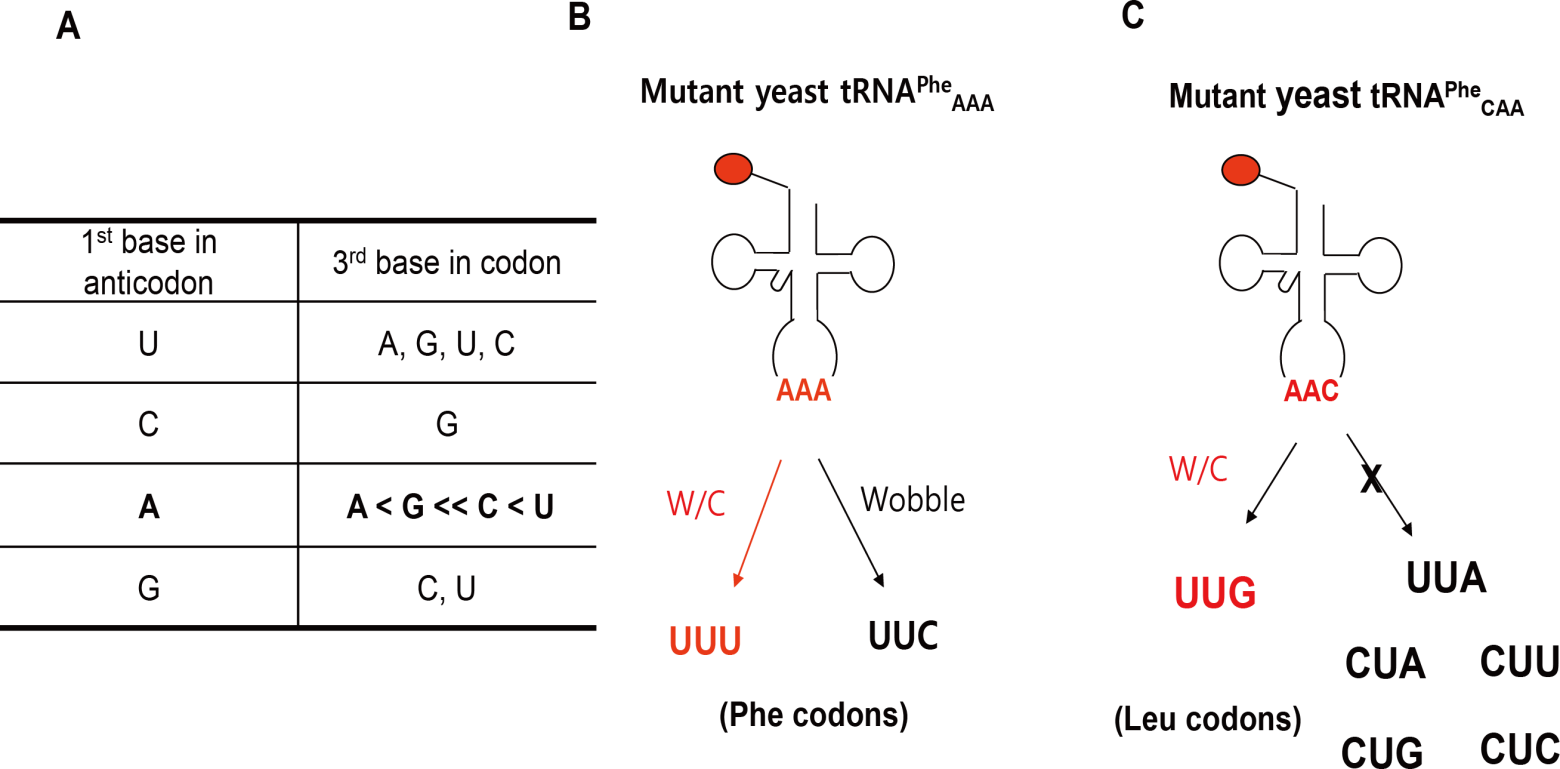
**Extended wobble rules (A).** Mutant ytRNA^Phe^_AAA_ recognizing UUU and UUC Phe codons by Watson-Crick (W/C) base pairing and wobble base pairing, respectively (B). Mutant ytRNA^Phe^_CAA_ recognizing UUG Leu codon by W/C base pairing but none of other Leu codons (C).

We reasoned that misincorporation of 2Nal at the UUC codon resulted from the recognition of UUC codons by the AAA anticodon of ytRNA^Phe^_AAA_. According to Crick’s wobble rule proposed in 1966 [29], the base A in the first position of the anticodon can recognize only the base U in the third position of the codon. Therefore, the UUC codon should not be recognized by the AAA anticodon. The discrepancy between the experimental results and Crick’s wobble rule may be explained by the expanded wobble rule proposed by Lim and Curran in 2001 [30]. The expanded wobble rule is based on new experimental findings [30–37] and stereochemical modeling [38–41] of codon-anticodon interactions. According to the expanded wobble rule (Fig 2A), A in the first position of the anticodon can recognize all four bases in the third position in the codon. The base A in the first position of the anticodon favors bases in the order U > C > G > A (Fig 2B), consistent with the codon-biased incorporation of 2Nal observed in this work.

### Incorporation of 2Nal into UUG Leu codons

**Fig 3:**
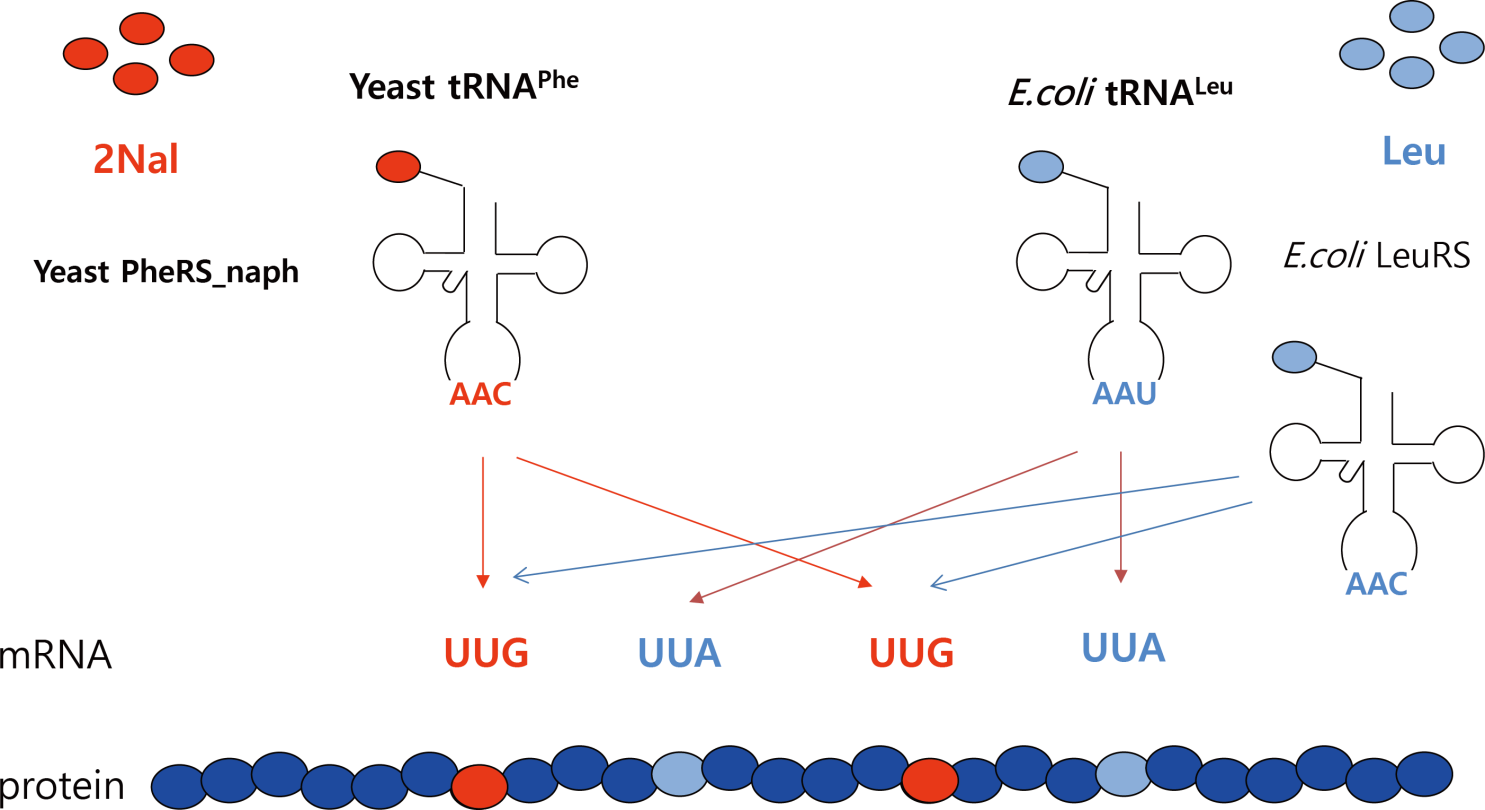
Scheme of 2Nal incorporation into multiple UUG Leu codons based on forced ambiguity of the Leu codons. *E*. *coli* leucyl-tRNA synthetase (LeuRS) charges Leu into its cognate tRNA^Leu^_s_ containing UAA anticodon (*E*. *coli* tRNA^Leu^_UAA_) and CAA anticodon (*E*. *coli* tRNA^Leu^_CAA_). Leu charged into *E*. *coli* tRNA^Leu^_UAA_ is incorporated into multiple UUA Leu codon sites of a target protein. The yPheRS_naph charges 2Nal into ytRNA^Phe^ containing CAA anticodon (ytRNA^Phe^_CAA_). Then, 2Nal is incorporated into multiple UUG Leu codons. According to the (extended) wobble rules, ytRNA^Phe^_CAA_ and *E*. *coli* tRNA^Leu^_UAA_ do not recognize UUA and UUG, respectively. UUG Leu codons can also be recognized by Leu-charged *E*. *coli* tRNA^Leu^_CAA_ resulting in partial occupancy of UUG codons by Leu.

According to the expanded wobble rules, the base C in the first position of the anticodon will recognize only the base G in the third position of the codon (Fig 2A). Therefore, we hypothesized that ytRNA^Phe^_CAA_ (containing the modified CAA anticodon) would selectively recognize UUG codons (Figs 2C and 3). Endogenous *E*. *coli* leucyl-tRNAs recognize all six Leu codons. One *E*. *coli* leucyl-tRNA containing the UAA anticodon (*E*. *coli* tRNA^Leu^_UAA_) recognizes UUA codons via Watson-Crick base pairing, but not UUG codons (Fig 3). In order to test this hypothesis of forced ambiguity of the Leu codons, we mutated the AAA anticodon of ytRNA^Phe^_AAA_ [20] into CAA anticodon by PCR mutagenesis using pREP4_ytRNA^Phe^_AAA_ as a template. Then, mDHFR was expressed in MP [pQE16_mDHFR2_lacI_yPheRS_naph/pREP4_ytRNA^Phe^_CAA_] cells. mDHFR contains twenty Leu codons, of which six are UUG, two are UUA and twelve are CUN (N = A/T/G/C). The expression level of mDHFR was 2.9 mg/L.

**Fig 4:**
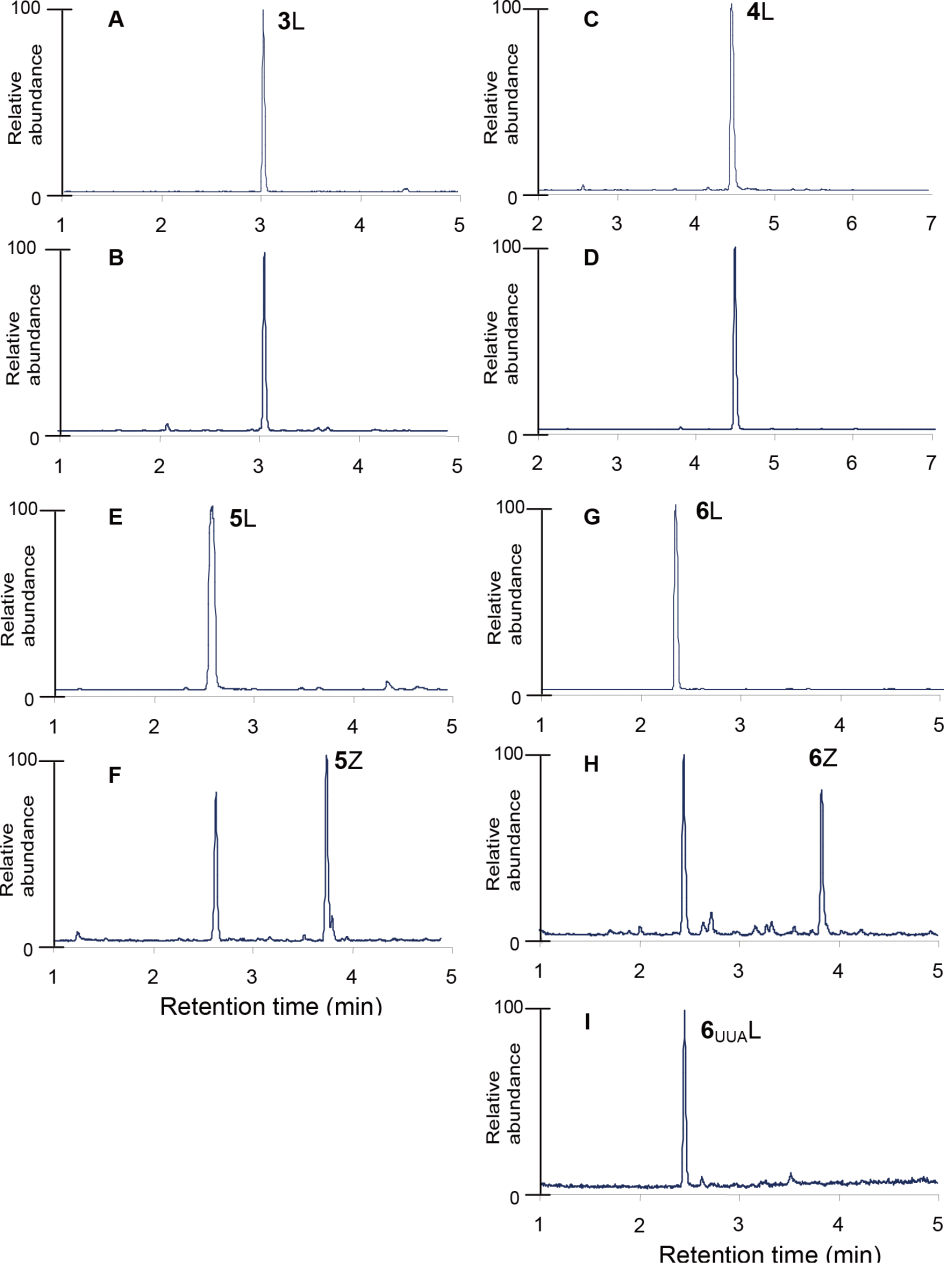
LC-MS chromatogram of tryptic digests of mDHFR. Peptide 1 (residues 165–180; L_CUU_L_CUC_PEYPGVL_CUC_SEVQEEK) contains three Leu residues encoded as CUU and CUC codons. Peptide 2 (residues 54–61; QNL_CUG_VIMGR) contains a Leu residue encoded as CUG codon. Peptide 3 (residues 62–70; L_CUU_IEQPEL_UUG_ASK) contains two Leu residues encoded as CUU and UUG codons. Peptide 4 (residues 99–105; SL_UUG_DDAL_UUA_R) contains two Leu residues encoded as UUG and UUA codons. Peptide 4_UUA_ is the same as Peptide 4 except both Leu residues are encoded as UUA codon. Peptide 1; 2; 3; 4; 4_UUA_ variants containing Leu and 2Nal were designated 1L and 1Z; 2L and 2Z; 3L and 3Z; 4L and 4Z; 4_UUA_L and 4_UUA_Z, respectively. These peptides were separated by LC and detected by MS. Unmodified mDHFR was synthesized in the absence of 2Nal in a Phe/Leu auxotrophic expression host (A, C, E, and G) in 2xYT media. Modified mDHFRs were synthesized in a Phe/Leu auxotrophic expression host outfitted with ytRNA^Phe^_CAA_ and yPheRS_naph (B, D, F, H, and I). The expression minimal media were supplemented with 17 amino acids (25 μg/mL), 1.25 μM Leu, 50 μM Phe, 50 μM Trp, and 3 mM 2Nal. No 1Z, 2Z, or 4_UUA_Z was detected by LC-MS analysis.

Occupancy of each Leu codon by various amino acids was determined by LC-MS analysis of tryptic digests of mDHFR expressed with and without 2Nal. We focused on four peptides. Peptide **1** (residues 165–180; L_CUU_L_CUC_PEYPGVL_CUC_SEVQEEK) contains three Leu residues, encoded as CUU and CUC codons. Peptide **2** (residues 54–61; QNL_CUG_VIMGR) contains a Leu residue, encoded as a CUG codon. Peptide **3** (residues 62–70; L_CUU_IEQPEL_UUG_ASK) contains two Leu residues, encoded as CUU and UUG codons. Peptide **4** (residues 99–105; SL_UUG_DDAL_UUA_R) contains two Leu residues, encoded as UUG and UUA codons. 2Nal was not detected at any CUN codon in Peptide **1** and **2** (Fig 4A–4D). However, 50% of the UUG codons in Peptide **3** and **4** were occupied by 2Nal (Fig 4E–4H). In order to determine UUA codon occupancy by 2Nal, Peptide **4**_UUA_ was tested. Peptide **4**_UUA_ is the same as Peptide **4** except both Leu residues are encoded as UUA codons. Since the Peptide **4**_UUA_ variant containing 2Nal was not detected, we concluded that 2Nal incorporation is highly specific to the UUG codon.

However, this strategy achieved only moderate UUG codon occupancy (~50%) by 2Nal (Fig 4E–4H), because UUG codon was partly occupied by Leu. Since *E*. *coli* leucyl-tRNA containing CAA anticodon (*E*. *coli* tRNA^Leu^_CAA_) can also recognize UUG codons (Fig 3), 2Nal-charged ytRNA^Phe^_CAA_ should competes against Leu-charged *E*. *coli* tNRA^Leu^_CAA_. Considering that the efficiency of amber codon suppression has been greatly improved in the past decade since its development, the occupancy of UUG codon by 2Nal is expected to increase in the future. For instance, an *E*. *coli* mutant deficient of *E*. *coli* tRNA^Leu^_CAA_ was previously reported [42]. If this mutant is further developed as an expression host for 2Nal incorporation, UUG codon occupancy by Leu would be eliminated, resulting in high fidelity incorporation of 2Nal at UUG codons. Furthermore, even partial incorporation of a noncanonical amino acid into programmed multiple sites would be useful for many applications, in particular, engineering protein-based biomaterials.

### Eliminated misincorporation of 2Nal into unwanted Leu codons

**Fig 5:**
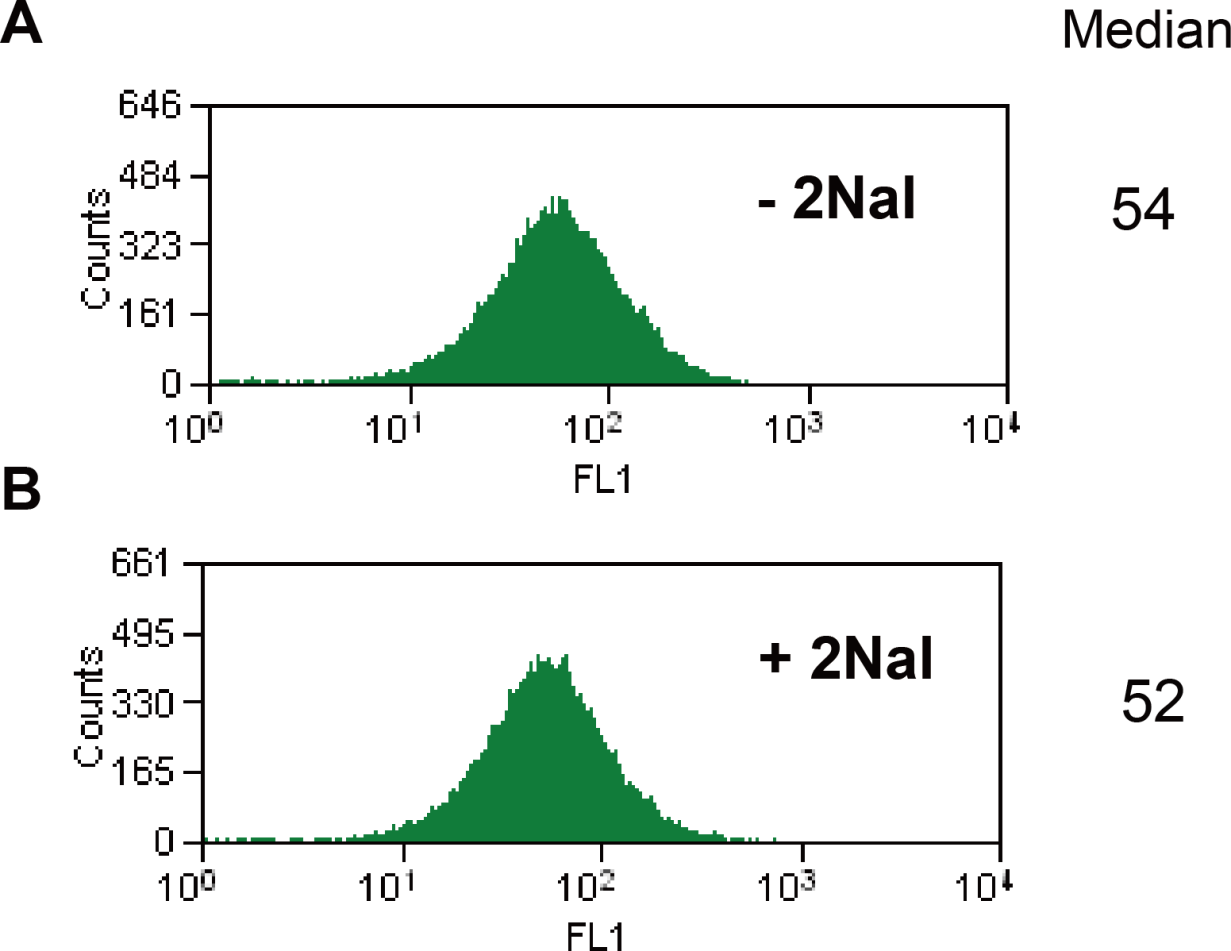
Fluorescence intensities of cells expressing GFP3. GFP3 was expressed in MPC390 expression hosts outfitted with yPheRS_naph and ytRNA^Phe^_CAA_ in minimal medium supplemented with 17 amino acids, 1.25 μM Leu, 5.0 μM Phe, 50 μM Trp, and no 2Nal (A); 3 mM 2Nal (B).

Next, we evaluated misincorporation of 2Nal at unwanted Leu codons using a GFP variant. We already showed that misincorporation of 2Nal at unwanted UUC Phe codons in GFP6 led to a 10-fold reduction in fluorescence of cells, even though there are no UUU codons in GFP6 (Fig 1A and 1B). Similar to GFP6, we used GFP3 containing twenty-three Leu codons, of which there were no UUG, four UUA, and nineteen CUN (N = A/T/G/C) codons. In order to investigate the effect of misincorporation of 2Nal at unwanted sites (Leu codons other than UUG), GFP3 was expressed in the *E*. *coli* strain MP [pQE9_GFP3_lacI_yPheRS_naph/pREP4_ytRNA^Phe^_CAA_]. The fluorescence intensities of cells expressing GFP3 without 2Nal or with 2Nal were compared (Fig 5A and 5B). There was no detectable difference in the fluorescence of cells prepared under these two conditions, implying the absence of significant misincorporation of 2Nal at Leu codons other than UUG.

### Codon occupancy depending on the anticodon of ytRNA^Phe^

As the first base in the anticodon of ytRNA^Phe^ changed from A to C, 2Nal assignment was accordingly changed from UUU to UUG. As an extension, we compared the UUU or UAG codon occupancy according to the anticodon of ytRNA^Phe^. First, UUU codon occupancy by tRNA^Phe^ containing AAA (ytRNA^Phe^_AAA_) was obtained by N-terminal sequencing of purified GFP6 variant containing a UUU Phe codon at position 2 (GFP6 (2UUU)). DHF [pQE9_GFP6_lacI_yPheRS_naph/pREP4_ytRNA^Phe^_AAA_] cells were induced to express GFP6 (2UUU) in MM18_FW medium supplemented with 50 μM Trp and 3 mM 2Nal, 80% and 20% of the UUU codon at the 2^nd^ position of GFP6 (UUU) were decoded as 2Nal and Phe, respectively, but Trp was not detected at this position (Table 1).

**Table 1:** Occupancy of UUU and UAG codons by various amino acids.

Codon	ytRNA	Occupancy of codon (%)^b^		
		2Nal	Phe	Lys
**UUU**^a^	**ytRNA**^**Phe**^_**AAA**_	80	20	ND^c^
**UUU**	**ytRNA**^**Phe**^_**GAA**_	92	8	ND
**UAG**^d^	**ytRNA**^**Phe**^_**CUA_UG**_	98	ND	2

^a^The second position in the amino acid sequence of GFP6.

^b^UUU and UAG codon occupancy was determined by N-terminal protein sequencing and MALDI-TOF MS analysis, respectively.

^c^Not detected.

^d^The 38^th^ position in the amino acid sequence of mDHFR_38Am.

**Fig 6:**
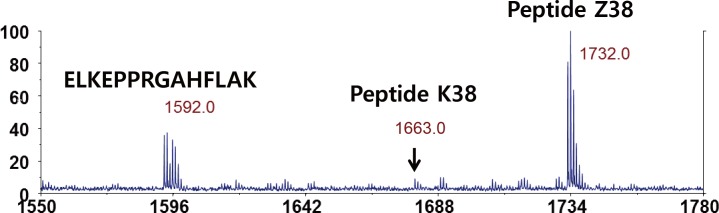
MALDI-TOF spectrum of tryptic digests of mDHFR_38Am. Peptide Z38 (residues 26–39; NGDLPWPPLRNEZK; Z indicates 2Nal) contains an amber codon at the 38^th^ position. Peptide K38 (residues 26–39; NGDLPWPPLRNEKK; K indicates Lys) contains Lys at the 38^th^ position. Another tryptic digest (residues 85–98; ELKEPPRGAHFLAK) contains Phe.

With an appropriate tRNA, the selective yPheRS_naph variant can be used for residue- and single-site-specific incorporation of 2Nal into proteins. In order to realize residue-specific incorporation of 2Nal, DHF [pQE9_GFP6 (2UUU)_lacI_yPheRS_naph/pREP4_ytRNA^Phe^_GAA_] expression hosts were induced to express GFP6 (2UUU) in minimal medium supplemented with 25 mg/mL 18 amino acids without Phe and Trp, 50 μM Phe, 50 μM Trp, and 3 mM 2Nal. N-terminal sequencing of the purified GFP6 (2UUU) showed that 92% of position 2 was occupied by 2Nal (Table 1), slightly higher than the 80% occupancy achieved by multiple-site-specific incorporation. The enhanced 2Nal incorporation may be a consequence of the known 12-fold higher aminoacylation rate for ytRNA^Phe^_GAA_ by yPheRS as compared to ytRNA^Phe^_AAA_ [43]. Site-specific incorporation of 2Nal into mDHFR_38Am, the mDHFR variant containing an amber codon at the 38^th^ position, was achieved by AFWK [pQE16_mDHFR_38Am_yPheRS_naph/pREP4_ytRNA^Phe^_CUA_UG_] in minimal medium supplemented with 25 μg/mL 17 amino acids (MM17_FWK), 50 μM Phe, 50 μM Trp, 50 μM Lys, and 3 mM 2Nal. The pQE16_mDHFR_38Am_yPheRS_naph plasmid was generated by replacing a yPheRS_T415A gene in pQE16_mDHFR_38Am_yPheRS_T415A plasmid [44] with a yPheRS_naph gene using standard molecular cloning techniques. Matrix-assisted laser desorption ionization-time of flight (MALDI-TOF) MS analysis of tryptic digests of mDHFR_38Am revealed that 2Nal was dominant at the amber site (Fig 6 and Table 1). Neither Trp nor Phe was detected, confirming the high selectivity of yPheRS_naph toward 2Nal.

## Conclusions

In this study, we developed a strategy to incorporate a noncanonical amino acid into multiple sites in a site-specific manner based on forced ambiguity of the Leu codons. Misincorporation of 2Nal at unwanted sites resulting from the less selective codon recognition of the AAA anticodon of ytRNA^Phe^ was overcome by use of the more codon-selective ytRNA^Phe^_CAA_. The CAA anticodon of ytRNA^Phe^_CAA_ completely discriminates UUG codon from the other five Leu codons. When both yPheRS_naph and ytRNA^Phe^_CAA_ were overexpressed in *E*. *coli* expression hosts, 50% of UUG codon sites were occupied by 2Nal, but no other Leu codon sites were occupied by 2Nal. Combined use of yPheRS_naph and the codon-selective ytRNA^Phe^_CAA_ realized multiple-site-specific incorporation of 2Nal into proteins. Furthermore, for the first time, these results successfully demonstrated that the concept of forced ambiguity of the genetic code is not limited to degenerate Phe codons, but can be generalized to other degenerate codons. Although the incorporation level of 2Nal at UUG codons was moderate (about 50%) due to UUG codon recognition by endogenous *E*. *coli* tRNA^Leu^_CAA_, we are working to improve the level of incorporation of 2Nal at programmed UUG sites using an *E*. *coli* mutant deficient of *E*. *coli* tRNA^Leu^_CAA_. Since the technique and strategy described here are very general, they would be applicable to incorporation of another noncanonical amino acid into a target protein when an appropriate orthogonal pair of the noncanonical amino acid is available.

## Materials and Methods

### Materials

Restriction enzymes were purchased from New England Biolabs (Beverly, MA, USA). Quikchange mutagenesis kits were purchased from Stratagene (La Jolla, CA, USA). Plasmid pREP4 and nickel-nitrilotriacetic acid affinity columns and were purchased from Qiagen (Valencia, CA, USA). 2Nal was obtained from Chem-Impex (Wood Dale, IL, USA). DNA primers were obtained from Operon Technologies (Huntsville, AL, USA) and Integrated DNA Technologies (Coralville, IA, USA). The reagents were purchased from commercial suppliers and used without further purification unless otherwise indicated.

### Preparation of *E*. *coli* expression hosts

A Phe/Trp double auxotrophic strain (AFW) and Phe/Trp/Lys triple auxotrophic strain (AFWK) were previously reported [44]. The Phe/Leu double auxotrophic strain, MPC390 (*leuB6*(*Am*), *PheA18*::*Tn10*), was supplied from the *E*. *coli* Genetic Stock Center (CGSC) at Yale University. A Phe auxotrophic derivative of DH10B (Stratagene) *E*. *coli* strain was generated by chemical mutagenesis [45] and replica plating. DH10B *E*. *coli* mutants were subjected to replica plating on minimal medium agar plates containing either all twenty amino acids or nineteen amino acids without Phe. An *E*. *coli* mutant that could not grow on minimal medium agar plates without Phe was selected as a Phe auxotrophic derivative of DH10B, designated DHF.

### Construction of Plasmids and Expression Hosts for Incorporation of 2Nal at Phe Codons

Construction of several GFP variants used in this study was previously reported [26]. An EGFP variant (GFP3) [46] has excitation maximum at 488 nm suitable for FACS analysis. A GFP variant (GFP6) was constructed by replacing all UUC Phe codons and one CUG Leu codon (at position 64) with UUU codons. A GFP variant (GFP3_WC) contains 12 Phe residues encoded as only UUC codons. In order to determine Phe codon occupancy by various amino acids, the AGA (Arg) codon in the second position of GFP6 was replaced by UUU or UUC Phe codon by PCR mutagenesis to generate GFP6(2UUU) or GFP6(2UUC), respectively [26]. Then, various *E*. *coli* host cells expressing either mDHFR or GFP variant were prepared using plasmids commercially available (Qiagen) or constructed previously [26]. Both pQE16_mDHFR_yPheRS (T415G) and pQE16_mDHFR_yPheRS naph were co-transformed with pREP4_ytRNA^Phe^_AAA_ into AFW competent cells to generate AFW [pQE16_mDHFR_yPheRS (T415G)/pREP4_ytRNA^Phe^_AAA_] and AFW [pQE16_mDHFR_yPheRS_naph/pREP4_ytRNA^Phe^_AAA_], respectively. In order to express intact mDHFR, pQE16 (Qiagen) and pREP4 plasmids were co-transformed into AFW competent cells to generate AFW [pQE16/pREP4]. Both pQE9_GFP6_lacI_yPheRS_naph and pQE9_GFP3_WC_lacI_yPheRS_naph were transformed into DHF_AAA electrocompetent cells to construct DHF [pQE9_GFP6_lacI_yPheRS_naph/pREP4_ytRNA^Phe^_AAA_] and DHF [pQE9_GFP3_WC_lacI_yPheRS_naph/pREP4_ytRNA^Phe^_AAA], respectively. Both pQE9_GFP6 (2UUU)_lacI_SD_yPheRS_naph and pQE9_GFP6 (2UUC)_lacI_SD_yPheRS_naph were transformed into DHF_AAA electrocompetent cells to construct DHF [pQE9_GFP6 (2UUU)_lacI_SD_yPheRS_naph/pREP4_ytRNA^Phe^_AAA_] and DHF [pQE9_GFP6 (2UUC)_lacI_SD_yPheRS_naph/pREP4_ytRNA^Phe^_AAA_].

### Construction of Plasmids and Expression Hosts for Incorporation of 2Nal at Leu Codons

The AAA anticodon of ytRNA^Phe^_AAA_ was mutated to CAA by PCR mutagenesis using pREP4_ytRNA^Phe^_AAA as a template to yield pREP4_ytRNA^Phe^_CAA_. The expression cassette of mDHFR was excised from pQE16 (Qiagen) by digestion with *Aat*II and *Nhe*I and inserted into pQE9_GFP6_lacI_yPheRS_naph [26] between the *Aat*II and *Nhe*I sites to generate pQE16_mDHFR_lacI_yPheRS_naph. In order to increase the number of Leu residues encoded as UUG, UUC and UUU Phe codons in position 38 and 95 of mDHFR were changed to UUG by PCR mutagenesis reactions using pQE16_mDHFR_lacI_yPheRS_naph as a template to generate pQE16_mDHFR2_lacI_yPheRS_naph. PCR mutagenesis reaction was performed to mutate UUG to UUA at position 100 of mDHFR2 to yield pQE16_mDHFR2 (100UUA)_lacI_yPheRS_naph. Either pQE16_mDHFR2_lacI_yPheRS_naph or pQE16_mDHFR2 (100UUA)_lacI_yPheRS_naph was co-transformed with ytRNA^Phe^_CAA_ into MPC390 competent cells to yield MP [pQE16_mDHFR2_lacI_yPheRS_naph/pREP4_ytRNA^Phe^_CAA_] or [pQE16_mDHFR2 (100UUA)_lacI_yPheRS_naph/pREP4_ytRNA^Phe^_CAA_], respectively. In order to express intact mDHFR, pQE16 (Qiagen) and pREP4 were co-transformed into MPC390 competent cells to generate MP [pQE16/pREP4]. pQE9_GFP6_lacI_yPheRS_naph was co-transformed with ytRNA^Phe^_CAA_ into MPC390 competent cells to yield MP [pQE9_ GFP6_lacI_yPheRS_naph /pREP4_ytRNA^Phe^_CAA_].

### Expression of mDHFR Variants and GFP Variants *In Vivo*

AFW and AFWK expression strains were co-transformed with pQE plasmid variants and pREP4 plasmid mutants. The strains were incubated in M9 minimal medium containing 0.4 wt % glucose, 35 mg/L thiamin, 1 mM MgSO_4_, 1 mM CaCl_2_, 20 amino acids (at 25 mg/L), 35 mg/L kanamycin, and 200 mg/L ampicillin. The expression strains were cultured for overnight, and were diluted 20-fold in fresh M9 minimal medium and incubate at 37°C. The cells were harvested when grown were reached OD_600_ = 0.8–1.0, and washed twice with cold 0.9% NaCl. The cells were resuspended in fresh M9 minimal medium supplemented with 18 amino acids (25 μg/mL), and the indicated concentrations of Phe, Trp, and 2Nal. The GFP expression was induced by addition of 1 mM isopropyl-β-D-thiogalactopyranoside (IPTG). After induced at 30°C for 4 hours, cells were harvested and either kept at -80°C or subjected to fluorescence measurement according to the procedures described earlier [26]. Whole cell lysates were analyzed by SDS-PAGE. Due to slow growth of DHF and MPC390 expression hosts co-transformed with pQE plasmid variants and pREP4 plasmid variants, transformants were grown in 2xYT medium to prepare glycerol stocks first. Then glycerol stocks were inoculated into minimal medium supplemented with 20 amino acids (at 25 mg/L) and incubated overnight at 37°C. The remaining steps were similar to those for AF and AFWK expression hosts.

### Flow cytometric analysis

When OD_600_ of DHF or MP900 cells expressing a GFP variant reached 0.6, the cells were washed twice with 0.9% NaCl solution. Then, the cells were resuspended with 20 mL of minimal medium supplemented with an appropriate amount of amino acids. The expression of a GFP variant was induced with 1 mM IPTG. After 3 hrs, 1 mL of the culture was collected, and washed twice with 0.5 mL of PBS (pH 7.4). 100 μL of cells were diluted with 3 mL of distilled water. Fluorescence intensities of the cells were analyzed by a MoFlo cell sorter. At least 20,000 events were collected in each measurement. Data were analyzed with Summit software (DakoCytomation).

### Quantitative Analysis of Codon Occupancy

Quantitative analysis of codon occupancy was performed by either N-terminal protein sequencing or LC-MS analysis of tryptic digests. The GFP6 (2UUU) and GFP6 (UUC) variants were expressed in minimal medium and purified by Ni-NTA affinity chromatography according to the manufacturer’s protocol (Qiagen) under denaturing conditions. The purified GFP variants were subjected to N-terminal protein sequencing using a 492 cLC Procise^®^ protein micro-sequencer (Applied Biosystems, Foster City, CA). Occupancy of Phe codons in mDHFR and Leu codons in GFP was determined by LC-MS analysis. mDHFR expressed in minimal medium were subjected to purification via Ni-NTA affinity chromatography according to the manufacturer’s protocol (Qiagen) under denaturing conditions. After purification, expression levels of GFP and mDHFR were determined by UV absorbance at 280 nm using a calculated extinction coefficient of 20,010 cm^-1^ M^-1^ and 24,750 cm^-1^ M^-1^, respectively. The purified proteins were concentrated by ultrafiltration (Millipore). 10 μL of the concentrate was diluted into 90 μL of 75 mM (NH_4_)_2_CO_3_ solution and then 1 μL of modified trypsin (Promega, 0.2 μg/μL) was added. Reaction was carried out for 2–4 hrs at 37°C and quenched by addition of 13 μL of 5% trifluoroacetic acid (TFA) solution. The solution was then directly subjected to LC-MS analysis conducted on a LCT Premier XE MICROMASS MS system (MS Technologies, Montgomery Village, MD) with Acquity UPLCTM system (Waters, Milford, MA). Tryptic digests were separated by Acquity BEH300 C18 column (1.7 μm, 300 Å, 2.1 x 50mm) using a gradient of 5–95% of solvent B (90% of acetonitrile/10% of 0.1% formic acid solution) and solvent A (2% of acetonitrile/98% of 0.1% formic acid solution) in 10 min. The column eluent was transferred to the electrospray source and mass spectra were recorded. MALDI-TOF MS analysis of tryptic digests of mDHFR was performed as described previously [20].
